# Can a mindfulness-informed intervention reduce aggressive behaviour in people with intellectual disabilities? Protocol for a feasibility study

**DOI:** 10.1186/s40814-016-0098-3

**Published:** 2016-09-20

**Authors:** Gemma Maria Griffith, Robert Jones, Richard Patrick Hastings, Rebecca S. Crane, Judith Roberts, Jonathan Williams, Lucy Bryning, Zoe Hoare, Rhiannon Tudor Edwards

**Affiliations:** 1 Centre for Mindfulness Research and Practice (CMRP), Bangor University, Bangor, UK; 2 School of Psychology, Bangor University, Brigantia Building, Penrhalt Road, Bangor, LL57 2AS UK; 3 Centre for Educational Development, Appraisal and Research: (CEDAR) University of Warwick, Coventry, UK; 4 Denbighshire Complex Disabilities Team, North Wales, North Wales, UK; 5 Centre for Health Economics and Medicines Evaluation (CHEME), Bangor University, Bangor, UK; 6 North Wales Organisation for Randomised Trials in Health (NWORTH), Bangor University, Bangor, UK

**Keywords:** Intellectual disability, Mindfulness-based, Aggressive behaviour, Feasibility study, UMAA-LD, Costs

## Abstract

**Background:**

Approximately 10–20 % of adults with intellectual disabilities engage in challenging behaviours such as aggression, destructiveness, and self-injury, which are often accompanied by feelings of anger. The inability to manage anger can reduce quality of life. For example, aggression is a strong predictor of out-of-area placements and is a risk variable for abuse. Recent research suggests that mindfulness-based therapies (specifically, Singh’s Soles of the Feet meditation) can help people with intellectual disabilities manage angry emotions, with resultant reductions in challenging behaviour. However, previous research has been single-case design studies, and no group studies have been published with people with intellectual disabilities and aggressive behaviour.

**Methods/design:**

For this feasibility study, a UK protocol will be developed for use by health professionals within National Health Service (NHS) Intellectual Disability (ID) teams, based upon Singh’s Soles of the Feet manual. Twenty adults with intellectual disabilities and identified problems with anger control will be recruited and six sessions will be delivered by a trained ID clinician. The study will monitor participant’s aggressive behaviour, health-related quality of life, anxiety, depression, and use of support services (medication, hospital appointments etc.). These will be measured at three time points: (1) Baseline (within 2 weeks prior to the first session of the intervention), (2) 2 months post-baseline, and (3) 6 months post-baseline. Qualitative interviews will be conducted with participants, their carers, and the therapists who delivered the intervention. In order to help design an economic evaluation alongside a future full trial, we will cost the intervention and test the acceptability and validity of health economics measures to record resource use and health-related quality of life outcomes.

**Discussion:**

The data from this study will inform the feasibility of the project protocol and intervention, which will help develop future research and to determine whether a larger, randomised controlled trial with concurrent economic evaluation is feasible.

**Trial registration:**

UKCERN: 16743.

## Background

In the UK, 2 % of adults and 3.5 % of children have an intellectual disability (ID) [1, 2]—defined as an IQ below 70, alongside adaptive behaviour deficits, and onset within childhood. Approximately 10–20 % of people with ID engage in challenging behaviour [3–7], and challenging behaviours are likely to persist over extended periods of time [8]. Severe challenging behaviour has a range of negative impacts, such as a lower quality of life, a negative impact on carer well-being [9, 10], reduced access to community services, and admission to intensive and specialist, and therefore costly, residential, or forensic services [11].

When interviewed, some adults with ID report that they find their own anger and challenging behaviour difficult to cope with and aversive and described feeling regretful after engaging in challenging behaviour [12–14]. Qualitative studies and a recent meta-synthesis have found that adults with ID are keen to learn how to self-manage anger [15].

Leading researchers on anger have called for creative and more effective interventions than the most common treatment methods such as cognitive behavioural therapy (CBT). CBT holds particular challenges for people with ID, as it requires high levels of language skills, and the ability to be introspective and self-aware, and yet does not explicitly address the development of such awareness [16].

There is an ongoing debate in the field about how best to define the complex construct of mindfulness (see [17]). An oft-cited definition of mindfulness is ‘paying attention in a particular way, on purpose, in the present moment and non-judgementally ([18]. p.4)’. Mindfulness-Based Stress Reduction (MBSR) was originally developed in the late 1970s, and MBSR courses today closely follow the original format. MBSR courses are taught in groups, for around 2.5 h a week for 8 weeks, with a full day of silent meditation practice around the sixth week. Participants are given home practices of around 45 min to an hour each day [18].

Mindfulness has the potential to address a number of psychological processes and states that are relevant to the risk of anger and resultant challenging behaviour [16].

These include reduced emotional reactivity to a trigger by observing anger-related sensations in the body without attempting to avoid or act on them. Evidence suggests that mindfulness is particularly relevant to the reduction of negative emotional episodes [19] because rumination increases the emotion of anger [20]. Conversely, a non-judgemental view of one’s thoughts may help reduce the negative impact of ruminative patterns. The ability to self-monitor one’s own mood states is regarded as being central to anger regulation. To adjust an individual’s anger response, there needs to be an awareness that anger is present. Aggressive outbursts may become autotomized anger responses, with little awareness of conscious attention (for people both with and without ID). Mindfulness training enables awareness of the bodily, cognitive, and behavioural signs of anger and opens the possibility of autotomizing non-aggressive responses [16].

Mindfulness-based interventions (MBIs) are recommended by the National Institute for Health and Care Excellence (NICE) in the UK. Specifically, mindfulness-based cognitive therapy (MBCT) is recommended for recurrent depression [21], and Dialectical Behaviour Therapy for Borderline Personality Disorder [22]. Typical MBIs rely on complex language to reflect on the experience of meditation, which may be cognitively demanding for individuals with ID. Therefore, it is important to consider how to adjust approaches for this population. In the USA, Soles of the Feet (SoF) meditation has been developed as a way of making MBIs accessible for people with ID [23]. It has been successfully used in ID populations to self-manage anger and, at long-term follow-up, has resulted in reported reductions or total elimination of aggressive behaviours [24–28]. It has also been used to help reduce deviant sexual arousal [29], smoking [30], and health behaviours contributing to obesity [31].

A recent systematic review [32] found the most frequent objective of MBIs for individuals with ID was the reduction of aggressive behaviour (explored in 7/12 studies), and of these, all reported a reduction in aggressive behaviour. Although there were 12 studies in the Hwang and Kearney review, this represents just 22 participants as they were all single-case design studies.

The Singh SoF manual [23] was originally designed to help individuals with ID cope with anger and reduce aggressive behaviour [25]. Individuals are taught to recognise situations that are ‘triggers’ for their anger, then guided through the steps of the SoF meditation. These include breathing naturally without trying to respond to angry thoughts or emotions and then shifting attention to, and being mindful of, the soles of their feet (a neutral part of the body). This technique is tailored to the cognitive level of the individual and enables the regulation of emotional states and behaviours. Thus, it is a good match with current philosophical and policy perspectives on empowering the lives of people with ID [32–34].

Hwang and Kearney [32] noted that teaching SoF to people with ID (in the studies, they reviewed) was labour-intensive, which creates a problem with intervention accessibility for patients and providers alike. The authors called for group evaluation: “Diversifying intervention methods and research methodology is the key” (p. 325). The proposed study addresses both the issue of labour intensity and of group evaluation. First, we aim to develop an adapted SoF package: Using Mindfulness for Anger and Aggressive Behaviour with people with Learning Disabilities—Soles of the Feet (henceforth referred to as UMAA-LD SoF), that is delivered over six sessions by health professionals working in the UK National Health Service (NHS). This serves to create an intervention that is comparable or shorter in timescale to other developed clinical interventions for anger and aggressive behaviour in this population [35–38]. Second, we are utilising a group design and so taking a step up the ‘evidence hierarchy’ from the single-case designs used thus far with SoF with people with ID and aggressive behaviour. Third, it is important that researchers are able to measure levels of mindfulness within a population where intervention studies are being conducted. Existing measures of mindfulness such as the Five Facet Mindfulness Questionnaire [39] are likely to be too cognitively demanding for people with ID. Therefore, development and validation of a measure of mindfulness for this population will be undertaken as part of the UMAA-LD study.

Few studies evaluating MBIs consider the costs along with the possible clinical benefits [40]. In the original SoF evaluation [28], a benefit-cost analysis indicated significant cost savings following SoF delivery due to a reduction in staff absenteeism at work. The SoF intervention costs were not included in the analysis as they were absorbed into routine behaviour training costs. These savings may not be representative of the costs of delivery within a UK health care setting. In order to evaluate whether SoF is a cost-effective intervention, a full economic evaluation alongside a randomised controlled trial is necessary. We chose to conduct a feasibility study because we wanted to explore the practicality of the proposed intervention and methodology. Therefore, this feasibility study will contribute to the growing evidence base of using SoF for people with ID and anger and aggressive behaviour.

### Aims

The principal aims of this feasibility study are

1. To test the UMAA-LD SoF protocol and research process including delivery across a NHS site, referral pathways, number of eligible participants and carers, treatment fidelity, willingness of clinicians to recruit, questionnaire completion rates, the time taken to complete outcome and process measures, and acceptability and accessibility of the UMAA-LD SoF protocol
2. To develop and carry out a preliminary test of the reliability and validity of a Mindful Awareness for adults with an Intellectual Disability Scale (MAIDS)
3. To cost the UMAA-LD SoF intervention and evaluate the acceptability and validity of the health economic outcomes for a subsequent full economic evaluation
4. To develop and test the feasibility of a protocol for a full-scale effectiveness randomised control trial (RCT) with concurrent economic evaluation of the UMAA-LD SoF intervention

## Methods

### Design

A single-arm study with 20 participants at three time points of measurement: baseline, immediately after intervention (2 months post-baseline), and follow-up (6 months post-baseline). A sample size of 20 is feasible and economical whilst providing sufficiently precise estimates of variances for powering larger studies. The UMAA-LD SoF intervention will take place over six sessions, at a rate of one session per week.

A researcher will visit participants at all three time points to assist with completion of outcome measures. This will take place either in the participant’s home or a place of their choice (e.g. their local clinic).

### Participants

Twenty participants will be recruited for the study. Inclusion criteria are (1) over the age of 18 years, (2) with an ID (established through the administration of the Wechsler Abbreviated Scale of Intelligence 2nd Edition [41]) and Adaptive Behaviour Assessment System© (ABAS; 2^nd^ Edition [42]), (3) have clinically significant difficulty with anger control as assessed by their clinician, (4) able to give informed consent, and (5) has a family member or paid carer who has supported them for a minimum of 6 months, is available to participate in the treatment sessions, and who currently provides a minimum of 2-h support per week to the participant. Although language ability is not an inclusion criterion, if participants are able to give informed consent, then they are also likely to have the language level needed to participate in an UMAA-LD SoF intervention.

Exclusion criteria are (1) existing diagnosis of autism spectrum disorder (because of potential difficulties with more abstract concepts relating to mindfulness when ID and autism are co-morbid), (2) presence of mental health problems or behaviour that may prevent the participant from interacting with the carer or therapist or retaining information from the therapy (e.g., dementia, active psychosis), and (3) individuals who are currently receiving direct psychological intervention (e.g. relaxation training, DBT, CBT).

### Recruitment

The research team will meet with clinicians working in NHS ID teams prior to commencement and explain the study. If clinicians are willing to be involved in recruitment, they will be given a supply of study information packs, comprising a letter of invitation, a participant information sheet, a carer information sheet, a reply slip, and a Freepost return envelope addressed to the researcher. Clinicians will be required to explain to the person with ID that a follow-up telephone call can be made by the researcher should they agree to this.

If the person with ID agrees to a follow up telephone call, clinicians will contact the potential participant once by telephone to check if they have retained the study information pack. If the pack has been mislaid, the clinician will offer to send a second pack. Adults with ID are often supported by several different carers, working across several settings (e.g. a person’s home environment, day centre). This situation means there is a high likelihood of study packs going missing.

On receipt of reply slips with the name and contact details of the potential participant agreeing to contact by the researcher, the researcher will arrange an initial screening visit so that they can explain verbally to the person with ID and their carer what the study involves and so that informed consent can be sought. Further visits/telephone contact can be arranged should the potential participant require more time before making a decision.

### Procedure

An initial screening visit (by a trained researcher) will seek informed consent and confirm whether the participant meets the inclusion criteria. If the inclusion criteria are met, the timescale for the three time points of measurement will commence. (1) Baseline (within 2 weeks prior to the first session of the intervention), (2) 2 months post-baseline, and (3) 6 months post-baseline. Data will be collected during face-to-face sessions with the researcher. The carer will also be available to support the person with ID should this be requested/necessary. At the 6-month post-baseline visit, the researcher will conduct semi-structured one-to-one interviews with participants and carers who took part in the intervention.

### Soles of the Feet meditation: intervention

#### Development of materials

The research team consists of international experts in the field of MBIs (RC) and intellectual disabilities (RJ, RH, JW) who will collaborate on the development of all materials. One of the researchers (JW) has experience of using variations of the original SoF protocol in routine clinical practice in the NHS prior to the trial. JW’s prior clinical experience of using SoF mindfulness approaches with people with ID in community settings helped inform the development of the UMAA-LD SoF protocol, e.g. highlighting concepts which participants often found difficult, with suggestions about how to make these more accessible.

#### Development of the UMAA-LD SoF protocol

The core SoF meditations [23] will be used in the UMAA-LD SoF manual. Singh et al. [23] provides a detailed description of how to teach Soles of the Feet mediation in the original manual. There are three main SoF meditations, all follow the same broad therapist instructions but with a different emphasis on the evoked emotion. The steps outlined below demonstrate typical therapist guidance during a SoF meditation (step 2: adapted from [23]):

1. Sit or stand in a natural position with the soles of the feet flat on the floor.
2. Breathe naturally.
3. Think about an incident that made you feel angry.
4. Notice the angry thoughts and stay with them, just notice how your body feels, and any thoughts or emotions that are around.
5. Quickly shift the focus of your attention to the soles of your feet.
6. Now, wiggle your toes. Feel your socks on the soles of your feet, focus on the arches of your feet. Go to the heels of your feet.
7. Continue wiggling your toes.
8. Now, open your eyes.
9. Well done for doing this Soles of the Feet practice.

There are three SoF meditations, each one introduced to the participant in a particular order, each meditation is intended be repeated until the participant is able to follow the instructions with fluency. In published research, the training times for SoF were usually in two stages, the first, an intensive training period with a therapist, usually between 5 days and a week, followed by a longer period of self-practice (between 12 weeks and 2 years), often with an audio recording [32].

- *SoF meditation (1)* Teaching Soles of the Feet meditation begins with the therapist asking the client to think of a happy situation and then following the steps of the meditation (see above).
- *SoF meditation (2)* The participant then practises the same meditation, but this time whilst evoking a situation that make them feel angry.
- *SoF meditation (3)* The participant then practises the meditation whilst thinking about a ‘trigger’ situation that typically causes them to get angry. This is the ultimate aim of the intervention—that the participant is able to identify triggering situations and use Soles of the Feet meditation appropriately in situ, before they get angry.

Although the original, core SoF practices [23] will be used, there will also be adaptions to the current manual, the main difference is that psycho-educational components around anger and stress reactions in the body will be added. The UMAA-LD SoF protocol will be designed as an addendum to the existing manual, rather than a replacement for it. The main additions will be as follows:

1. *UMAA-LD SoF will be designed specifically for use in NHS settings.* The SoF manual [23] does not specify what content is covered on a session-by-session basis. The UMAA-LD SoF manual will provide specific outlines for each of six sessions. Each session will take up to 90 min, with the clinician pacing the session to meet participants’ needs. See Table 1 for a typical session format.
2. *UMAA-LD SoF will provide educational materials* suitable for people with ID that will focus on anger, aggression, and mindfulness. These will be used in sessions and also form a ‘pack’ that the participant can take home and read.
3. *UMAA-LD SoF sessions will require the participant (with carer support) to engage in short, daily home meditation practices each week.* The home practices given will follow the progression of the session. A CD/audio file that will have a recording of the Soles of the Feet meditation will be provided in a format best suited to the participant. These audio recordings of SoF practices will be specially designed for the ID population and will last for around 4–6 min each.

**Table 1:** Typical format for a UMAA-LD SoF session

Content of session	Approximate time frame (min)
Introducing the session	5–10
Review homework log	5–10
Psycho-educational component, i.e. what is anger? What is mindfulness?	20–30
Comfort break	5–10
Soles of the Feet meditation practice	20–30
Review of the session and home practice setting for next week	5–10

### Therapist training and supervision to deliver UMAA-LD SoF

All therapists will be trained clinical psychologists, supervised trainee psychologists, intellectual disability nurses or other NHS clinicians. The UMAA-LD SoF training was for a full day, co-delivered by clinicians (RJ and JW), and a mindfulness-based teacher (GG). During the training day, the manual was explained on a session-by-session basis and core SoF practices were demonstrated and explained, and therapists practiced delivering the SoF meditations in the training session. Therapists were encouraged to attend an MBSR class if they had not already done so and to sustain their personal mindfulness practice after the training and during delivery of SoF sessions. This is due to the importance of mindfulness being communicated through the therapists embodied practice during sessions. Therapists will receive two supervision sessions over the six sessions for each participant. At least one supervision session will be with a mindfulness-based teacher (GG) to discuss issues pertaining to the mindfulness elements of the intervention. The other supervision session will be provided by a senior ID clinician (RJ or JW) or by a clinician and researcher (JR).

### Mindfulness scale for people with ID

To address aim 2, the following methodology will be adhered to.

The MAIDS will be a self-report measure to elucidate mindfulness processes in relation to problem areas, such as anger. Development of items will be informed by three sources: (1) examination of existing mindfulness measures (no current measure exists for use with people with ID), (2) research on the development of self-report measures for people with ID, and (3) consultation with mindfulness and ID professionals. To date, a working version of MAIDS (consisting of ten items) has been developed. The MAIDS will be incorporated in the UMAA-LD SoF protocol to test its feasibility as an evaluation tool.

The MAIDS will be tested on the 20 participants in the intervention study, at all three time points. In addition, to help determine reliability and validity of the scale, 80 further participants with ID will be recruited to complete the MAIDS, the Modified Overt Aggression scale [43], Novoco Anger Scale [44], Glasgow Anxiety [45] and Glasgow Depression Scale [46] at a single time point. Participants will be recruited from various sources, including local ID groups and residential homes. Informed consent will be gained prior to collecting data and will be collected by researchers visiting the participants to assist where required.

### Measures

A researcher is responsible for collecting all feasibility data and outcome measures. The primary aim of these outcome measures is to test the suitability and practicality of the measures with adults with ID. Following the initial screening visit, where capacity to consent is assessed, a baseline visit will be arranged prior to the commencement of the intervention where demographic information on age, gender, and current residential status will be gathered. All of the feasibility outcome measures described below will be taken at the three time points of baseline, 2 months post-baseline, and 6 months post-baseline.

#### Feasibility process measures

To address aim 1, the following measures will be taken.

1. Screening and recruitment rates will be monitored.
2. The MAIDS to measure skills in using mindfulness to manage anger.
3. Data on adherence to mindfulness home practice.
4. Participant drop out from the feasibility study will be closely monitored and reasons gathered where possible.
5. An assessment of the completion rates of the questionnaires will be completed. This will encompass an analysis of any missing data patterns.
6. Information gleaned from the qualitative interviews will be used to inform understanding of the process.
7. Intervention fidelity will be monitored. Two out of six sessions with each participant will be audio recorded. One will be from the start of the intervention (sessions 1–3) and the other from sessions 4–6. The recordings will be evaluated using an adaptation of Singh’s Soles of the Feet monitoring form that evaluates adherence to the Soles of the Feet manual [23].
8. A clinical input questionnaire will capture the participants’ recent clinical input (e.g. from a behavioural specialist, any other direct psychological interventions) and be completed by the carer with the researcher during the consent visit, this is repeated during the baseline visit to identify any recent changes.

#### Demographic measures

Following the initial screening visit, where capacity to consent is assessed, a baseline visit will be arranged prior to the commencement of the intervention where demographic information on age, gender, and current residential status will be gathered. As part of the inclusion criteria, the presence of an ID will be confirmed at baseline using two measures: (1) Wechsler Abbreviated Scale of Interlligence-Second Edition [41] that measures IQ in adults and (2) Adaptive Behaviour Assessment System® (ABAS)—Second Edition [42] that is completed by the carer and measures abilities, skills and physical, and sensory impairments.

#### Measures (administered to the participant with ID)

All of the potential outcome measures described below will be taken at the three time points of baseline, 2 months post-baseline, and 6 months post-baseline.

All self-report measures used have been adapted for use with an ID population or have been evaluated by the research team and are likely to be appropriate measures for use with individuals with ID.

- *Novaco Anger Scale (NAS):* A 60-item scale exploring how an individual experiences anger, with three subscales: cognitive, arousal, and behavioural, that constitute a total score for anger disposition [44].
- *Provocation Inventory (PI):* A 25-item scale that identifies the kinds of situations that induce anger in particular individuals [47].
- *The Glasgow Depression Scale for adults with Learning Disabilities (GDS-LD):* A 20-item scale that asks how often depressive symptoms have occurred over the past week [46].
- *The Glasgow Anxiety Scale for adults with Intellectual Disabilities (GAS-ID):* A 27-item self-rating scale that assesses how often anxiety symptoms have occurred over the past week [45].
- *EQ-5D-Y (Youth version of the EQ-5D generic Health Related Quality of Life questionnaire: HRQoL):* This is a standardised and validated instrument on HRQoL and is intended for use with children and adolescents aged between 7 and 12 years old [48, 49]. There are currently no generic quality of life questionnaires adapted for people with ID.
- *CHU-9D (A paediatric generic preference based measure of HRQoL):* This is intended for use with children aged 7–17 years [50]. There are currently no generic quality of life questionnaires adapted for people with ID and challenging behaviour.
- *ICECAP-A (ICEpop CAPability measure for Adults):* A measure of capability for the general adult (18+) population for use in economic evaluation [51].

#### Potential outcome measures-proxy (carer) report

The primary outcome measure is the Modified Overt Aggression Scale (MOAS: [43]) measures four types of aggressive challenging behaviour over the past week (verbal, against objects, against self, against others) and measures both severity and frequency. The MOAS has good internal consistency and test-retest reliability (*α* = 0.75) for the rating of aggression. The MOAS will also be administered to the carer by the clinician delivering the intervention on a weekly basis.

Carers will also complete proxy versions of the EQ-5D-Y [48], CHU-9D [50], and ICECAP-A [51] so that their perception of the quality of life of the person they care for can be measured. In addition, the following measures will be utilised.

- *The Glasgow Depression Scale-Carer supplement (GDS-CS):* A 16-item scale that asks how often depression symptoms have occurred over the past week [46].
- *Anxiety, Depression, and Mood Scale (ADAMS):* As there is no equivalent of the GDS-CS41 for anxiety, the general anxiety scale of the ADAMS will be utilised as a proxy report measure of anxiety [52].
- *Client Service Receipt Inventory-European Version (CSRI-EU):* This has been used in previous ID research and examines the participant’s use of medication and health care, social care, day, and community services [53].

#### Qualitative interviews

Qualitative semi-structured one-to-one interviews with participants and carers will be conducted after all outcome data have been collected (6 months after baseline) focusing on the interview topics described below.

- *Participants:* How accessible/acceptable SoF meditation was, the impact upon their feelings and expressions of anger, their quality of life, adherence and perceived barriers to the intervention, use of the mindfulness practice audiofiles at home, whether they will continue to use SoF meditation, exploration of any relationship changes with their carer, and any future support needed.
- *Carers:* Perceived impact of the intervention on their offspring/client, their relationship with their client/ family member throughout the intervention, and exploring what, if any, impact it had on their own lives, both personally and as a carer.
- *UMAA-LD SoF therapists:* their experiences of mindfulness prior to training, their experience of being trained to use UMAA-LD SoF, experiences of delivering the UMAA-LD SoF, including the accessibility of the manual for themselves and clients and the perceived effects on participants and carers.

### Method of analysis

As this is a feasibility study, no hypothesis is to be tested, and no formal analysis of outcome variables will be made as the study is not powered for definitive analysis.

The primary measures of interest are patient recruitment, attrition, and response rate for questionnaires. Feasibility metrics (e.g. recruitment and retention rates, clinical characteristics, duration of the intervention, etc.) will be analysed first, together with adherence outcomes (patient acceptance and adherence to the intervention). The mean change from baseline together with associated variances will be calculated for all measures and will be presented as point estimates together with 95 % confidence intervals. An estimation of the precision of the means and variances will be made to inform the power calculation for the main RCT protocol. Framework analysis [54] is the planned approach to analysing the qualitative interview data.

Initial psychometric properties of the MAIDS will be evaluated using baseline data collection. Internal consistency for the MAIDS total score will be estimated in addition to initial convergent validity (association with baseline mental health measures).

#### Health economic evaluation

To address aim 3, the health economics component of the study will be conducted from a public sector multi-agency perspective [55]. As there is limited evidence of self-reported HRQoL and service use questionnaires being used in an ID population, the principle aim of the health economics evaluation will involve piloting the key outcome measures. A comparison will be made between the participants’ self-reported HRQoL and proxy ratings of HRQoL. Descriptive statistics will be compared with other study outcomes and available social norms. The correlation between study outcome measures will be assessed to explore the construct validity of measures in this population.

The intervention will be costed using micro-costing techniques that have previously been successfully used in costing MBIs and other complex psycho-social interventions [56]. Patterns of health care, social care, and other service use over the preceding four months will be explored and costed using national unit costs [57, 58]. A period of 4 months is sufficient for a representative picture of service use to be gauged, yet recent enough for the respondent to recall accurately the frequency and nature of contacts [59, 60]. As the intervention follow-up period is less than 1 year, it will not be necessary to discount costs [55].

#### Protocol for a full-scale effectiveness trial

To address aim 4, a full study would be considered feasible if (1) recruitment rate found was more than 50 %, (2) if the retention rate found was more than 50 % at final follow-up, and (3) if adherence rate was greater than 50 % for the completing participants.

### Ethics and consent

Ethical approval has been received from both Bangor University, and the NHS Research Ethics Committee (ref 15/WA/0213), and the study is registered with the UK Clinical Research Network Study portfolio—database no. 16743. Our protocol around assessing capacity to consent has been designed in accordance with the Mental Capacity Act [61] and ID best practice [62]. We will use developmentally appropriate information sheets and consent forms. It will be made clear to participants that they have the right to withdraw from the study at any time. Those unable to consent and those who do not consent will continue to receive treatment as usual from their service. Consent will be sought from carers separately.

## Discussion

The prevalence of aggressive behaviours among people with ID is further accentuated by limited access to evidence-based interventions. Leading ID researchers have called for creative and more effective interventions for anger [16].

Research in the field of ID shows the negative impact of challenging behaviour resulting from anger that can lead to a lower quality of life, a negative impact on carer well-being, reduced access to community services and admission to intensive and specialist, and therefore costly, residential, or forensic services [3, 9–11]. Therefore, within specialist ID NHS services and mainstream mental health services, there is a need to further develop clinical practice to support adults with ID with mental health problems, including anger and aggressive behaviour. There has been some work to adapt and evaluate CBT methods for anger (although few examples of high-quality research), but little focus on adapting other psychological therapies from mainstream services.

This feasibility study will examine an evidence-based intervention for anger in adults with ID and is anticipated to benefit participants directly by offering a valuable treatment approach where limited approaches exist currently. An intervention informed by mindfulness may also have other benefits with an ID population because it encourages the individual to develop an attitude of shared responsibility for their own mental health with consistent practice of self-management skills. Thus, the proposed UMAA-LD SoF intervention is potentially useful both as an early intervention measure for reduction in aggressive behaviour and also as a strategy for long-term management of anger and aggressive behaviour. Mindfulness training is also non-intrusive, an alternative to medication and promotes resilience and well-being. By developing familiarity with mindfulness and basic meditation techniques, adults with ID may also be better placed to access mainstream mindfulness classes. Adults with ID may (perhaps with the support of their carer) be empowered to manage other emotional problems (e.g. anxiety) using their new transferable mindfulness skills. Finally, reducing anger and associated challenging behaviour may contribute towards participants’ ability to engage with paid employment, improving their economic situation and providing meaningful activity.

In health economics evaluations, the NHS traditionally prefers a cost-utility approach to economic analysis, whilst in Public Health cost-benefit and cost-consequence analysis are considered to be more appropriate by NICE [63]. The UMAA-LD SoF intervention may result in benefits and possible cost savings that goes beyond the health service; therefore, a public sector multi-agency approach to economic analysis is likely to capture this wider potential impact of the intervention.

There will likely be variability in therapists’ previous experience of practicing or teaching mindfulness-based approaches. The UK Good Practice Guidelines for teachers of mindfulness-based programmes such as MBSR and MBCT stipulate that those delivering MBIs have personal experience of mindfulness practice [64]. However, due to practicalities of recruiting ID health professionals to deliver UMAA-LD SoF, we will aim for this but anticipate this will not always be possible. Those who do not have personal experience of mindfulness practice in advance will be offered support and encouragement to develop this. Although this intervention is mindfulness informed, it is not mindfulness-based, because it does not comply with UK good practice guidelines (although these were developed for teachers of the 8-week courses for the general public or clinical populations and are not intended to be specific for ID populations [64]). This aspect of the project will be closely monitored as part of the feasibility study, through the qualitative interviews with the therapists, as the personal practice of the teacher and how this is embodied could be important with an ID populations as it bypasses cognitive verbal channels.

We anticipate that the qualitative information will enhance the quantitative analysis and RCT protocol. Although preliminary data on the effectiveness of the intervention will be taken, it will not give insight into the mechanisms of change. This question will be explored in the qualitative data, with participants, their carers, and with clinicians. The qualitative data will also help identify any practical barriers or facilitators to participating in the intervention and any unanticipated positive or negative affects of the intervention.

Successful anger management intervention trials using RCT have been utilised for people with ID [38] allaying concerns expressed by some researchers about whether it is feasible to undertake RCT’s with people with ID [65]. This feasibility study will examine whether UMAA-LD SoF may be suitable for investigation as a large-scale RCT. First, all data collected will be used to make any final revisions to the UMAA-LD SoF protocol, if necessary. Secondly, if the feasibility study is promising, an intervention package and protocol for an RCT with concurrent economic evaluation will be developed. The intervention package will include a validated UMAA-LD SoF manual, a new mindfulness measure with initial psychometric data (MAIDS), a complete description of the intervention, clinician training method, method to measure treatment fidelity, and follow-up procedures. The protocol will draw on pilot data for recruitment pathways and inclusion and exclusion criteria. It will provide valuable information into the feasibility of key issues such as recruitment and acceptability of the UMAA-LD SoF protocol with participants, carers, and clinicians.
